# Analysis of antibody induction upon immunization with distinct NTS-DBL1α-domains of PfEMP1 from rosetting *Plasmodium falciparum* parasites

**DOI:** 10.1186/1475-2875-12-32

**Published:** 2013-01-24

**Authors:** Davide Angeletti, Letusa Albrecht, Mats Wahlgren, Kirsten Moll

**Affiliations:** 1 Department of Microbiology, Tumor and Cell Biology, Karolinska Institutet, Stockholm, SE-17177, Sweden; 2 Dep. Genética, Evolução e Bioagentes, UNICAMP, Instituto de Biologia, Cidade Universitaria Zeferino Vaz, Rua Monteiro Lobato 255, Campinas, SP, 6109, Brazil

**Keywords:** *Plasmodium falciparum*, Malaria, PfEMP1, DBL1α-domain, Rosetting, Subunit vaccine, Antibody titres, Epitopes, Cross-reactivity

## Abstract

**Background:**

Rosette-formation of *Plasmodium falciparum* parasitized erythrocytes is of importance in the development of severe malaria. The parasite-derived molecule PfEMP1 (*Plasmodium falciparum* erythrocyte membrane protein 1), central to rosetting, is suggested to be included in a multimeric vaccine targeting severe disease.

**Methods:**

Three recombinant NTS-DBL1α-domains of PfEMP1 were generated in *Escherichia coli*, purified and used for immunization of rats and goats. Antibody titres were determined in ELISA assays and responses were compared in-between different individual animals and species. Reactivity with the parasites was tested in live pRBC using FACS. B-cell epitopes prediction was carried out *in silico* and compared to the results obtained by peptide microarray. Screening for serological cross-reactivity with heterologous NTS-DBL1α variants was carried out by ELISA, peptide array and FACS on pRBC of different laboratory strains and patient isolates.

**Results:**

All three NTS-DBL1α-domains induced high titres of antibodies that were biologically active with no apparent difference between constructs covering slightly different parts of the DBL1α-sequence. The different animal species showed comparable titres of antibodies, while variations within individuals of the species could be observed.

Mapping of the recognized epitopes revealed that most parts of the molecule were able to induce an antibody response with a tendency for the N and C terminal parts of the molecule for slightly higher recognition. Important differences to the epitopes predicted were found as some of the most conserved parts of the DBL1α-domain contained the main epitopes for antibody reactivity. ELISA assays and peptide microarray demonstrated substantial cross-reactivity to heterologous variants, while binding to native PfEMP1 was observed only in few combinations on the pRBC surface, underlining that mainly internal, conserved and not surface exposed parts of the DBL1α-domain are responsible for this observation.

**Conclusion:**

Biologically active antibodies can be induced consistently, with high titres, in different animal species and the antibodies elicited by different constructs react with similar epitopes. Induced antibodies recognize epitopes localized in all subdomains of the DBL1α-sequence. Cross-reactivity between NTS-DBL1α-variants is common in ELISA, but rare with live pRBC emphasizing that also internal, conserved areas of PfEMP1 carry important highly immunogenic epitopes of the molecule.

## Background

Rosetting, the binding of parasitized to non-parasitized red blood cells (RBC), has been described as an important virulence factor of the *Plasmodium falciparum* parasite. Rosetting has been found associated with severe malaria in many studies in Africa [1-8], has been described to lead to microvascular obstruction [9,10] and has been suggested as one of the most important factors bringing about severe disease [11,12].

During rosetting, the parasite ligand *P. falciparum* erythrocyte membrane protein 1 (PfEMP1) binds serum proteins and receptors on the human RBC surface. So far, serum proteins, such as non-immune immunoglobulins, fibrinogen and albumin as well as blood group A and B antigen, heparan sulphate [13-18] and the complement receptor 1 (CR1) [19,20] have been identified to be involved in the rosetting phenomena.

The PfEMP1 protein family is the by far best characterized group of parasite ligands linked to the parasite’s capacity to cytoadhere [21-23] and rosette [19,24,25], however other molecules have been suggested to be involved in these adhesive events. PfEMP1 proteins share a common structure of tandemly arranged Duffy Binding Like domains (DBL) and Cysteine-rich InterDomain Regions (CIDR). PfEMP1 vary in size between 200–400 kDa and are encoded by a repertoire of around 60 *var* genes per genome [26] responsible for the antigenic variation at the pRBC surface [27-29].

The N-terminal NTS-DBL1α-domain of the PfEMP1 molecule is central in the binding event to host RBC [15,19,24,30]. To date, three different NTS-DBL1α-variants involved in rosetting have been analysed in detail: NTSDBL1α-R29^var1^[19], NTSDBL1α-PA^varO^[24] and NTSDBL1α-FCR3S1.2^var2^[25]; all three variants are encoded by group A *var* genes. This observation based on parasite laboratory strains is supported in *P. falciparum* patient isolates, where a correlation between rosetting and the transcription of group A *var* genes exists [31-34].

Although a central role of the variant PfEMP1 molecule in the acquisition of malaria protective antibodies has been underlined in a number of studies [35-45], few have specifically investigated anti-rosetting antibodies. There is the indication that antibodies able to disrupt rosettes are involved in protection against severe disease [1,2] and antibodies targeting domains involved in rosetting can promote the opsonization of the pRBC [46-48]. Further, polyclonal antibodies towards the rosette-associated DBL1α-domains have been shown to be able to disrupt rosettes of the homologous [19,24,25] and recently also of heterologous parasite strains [46], generating conflicting data whether epitopes exposed by rosetting pRBC are variant specific [49] or shared by parasites displaying a similar adhesive phenotype [46]. In addition, there is to date no information available about which epitopes are targeted by these antibodies and where they are located within the molecule. PfEMP1-variants linked to rosetting are, due to the strong association between rosetting and severe disease, promising vaccine candidates. The development of a vaccine based on a recombinant domain derived from PfEMP1 needs to be initiated with the detailed analysis of the vaccine-induced protective immune response in an animal model, even though immunological responses in such models are often only indicative of what will be observed in the human host.

This study reports the generation of antibodies against three NTS-DBL1α-domains in two different animal species, and the comparison of their antigenicity and serum titres induced by the antigens. Antibodies were found to be biologically active and were mapped for their specific epitopes in peptide microarrays. Detailed analysis of their capacity to cross-react with other DBL1α-variants was carried out both in regard to linear epitopes as well as epitopes displayed by the native protein on the pRBC surface.

## Methods

### Parasite cultures

Culture of *P. falciparum* laboratory clones/strains was carried out according to standard methods [50], while the protocol was slightly modified for patient isolates [51]. Seven different patient isolates, collected in Uganda [8] were used in this study (UKS111, UKS31, UKS221, UAS22, UKM62, UAM51 and UAM15). For the maintaining of the rosetting phenotype of FCR3S1.2, R29 and PAvarO enrichment with monoclonal antibodies was performed [24].

### Production of recombinant protein

Expression constructs of the three His-tagged NTS-DBL1α domains used here was performed as described [52]. For IT4*var*60 the expression was carried out in *Escherichia coli* Shuffle T7 express: bacteria were grown at 30°C till OD_600_ = 0.6 and subsequently induced with 0.4 mM IPTG for 20h at 16°C. Pelleted cells were first subjected to osmotic shock, as described [53], and subsequently lysed by sonication. The soluble part, containing the recombinant protein, was separated by centrifugation at 12,000 g for 15 min and subsequently purified.

For IT4*var*9 and PAvarO, BL21 (DE3) bacteria were grown till OD_600_ = 0.8. Culture was induced for 3 h at 37°C with 0.1 mM IPTG. Following induction the cells were lysed by sonication, crude inclusion bodies were pelleted upon centrifugation at 12,000g for 30 min and solubilized in denaturing solution (6M Guanidine HCl, 50mM Tris–HCl pH 8, 100mM NaCl, 10mM EDTA pH 8, 10 mM DTT) overnight at +4°C. The recombinant proteins were refolded by the method of rapid dilution: the protein solution was filtered and added dropwise to ice-cold refolding solution (200 mM Tris–HCl pH 8, 10mM EDTA pH 8, 0.6M L-arginine, 6.5 mM cysteamine, 3.7mM cystamine) to a final concentration of 0.2 mg/ml. Refolding was allowed to proceed at +4°C for 36 h.

The recombinant DBL1α-domains were then dialysed to remove the excess of arginine and EDTA and concentrated using Amicon Ultracel centrifugal filter units (Millipore). All proteins were purified by Immobilized Metal Affinity Chromatography over TALON Cobalt column (Clontech), eluted with 200 mM imidazole and further purified to homogeneity by size exclusion chromatography on a HiLoad 16/60 Superdex 75pg colum (GE-Healthcare).

### Generation of antibodies in goats and rats

Polyclonal antibodies were produced in goats commercially by Agrisera (Vännäs, Sweden). Animals were immunized four times at one-month intervals with 200 μg of protein; the protein was emulsified in Freund’s complete for the first immunization and incomplete adjuvant for the following three immunizations. Final bleeding was carried out two weeks after the last immunization. In addition, α-NTS-DBL1α-sera generated in earlier studies [25,54] were used. Briefly, sera against the NTS-DBL1α_S1.2var1, 3d7var5.2, PAvarO_ (mixed) and NTS-DBL1α_S1.2var1, 3d7var5.2, PAvarO_ (sequential) were generated in rats by immunization with SFV-particles on day 0, 30 and 60 (1×10^8^ particles/rat) and with recombinant protein emulsified in Montanide ISO 720 (Seppic, France) on day 90 (100 μg protein/rat) subcutaneously [54]. Further, rat sera against the NTS-DBL1α_IT4*var*60_[25] and the NTS-DBL1α _T4*var*9_ were generated by immunizing rats trice with 100μg his-tagged recombinant NTS-DBL1-protein emulsified in Freund’s complete (first immunization), respectively incomplete adjuvant (second, third immunization).

### Ethics statement

The animal studies were approved by the Swedish Board of Agriculture (permission rats: N237/07, N103/10; goats: A37/10).

### ELISA

Reactivity of the generated antibodies with recombinant protein was tested in ELISA assays as described [30]; briefly plates were coated with 2 μg/ml protein overnight, subsequently blocked and thereafter incubated with 100 μl antibody containing solution in serial two-fold dilutions between 10 to 0.001 mg/ml. Reactivity was visualized using an ALP-coupled antibody against the corresponding species; pre-immune serum (rat) or pre-immune goat IgG was used as a control for background binding in all experiments.

### Analysis of surface reactivity of pRBC by flow cytometry

Analysis of surface reactivity was carried out as described [52], briefly, pRBCs of ≈ 24-30h p.i. were incubated with goat IgGs (final concentration 10 μg/ml), or rat sera (final dilution 1:10). Non-immune goat IgG, respectively rat pre-serum in the same concentration was used as control in all experiments; reactivity was visualized with an ALEXA488-coupled, species specific secondary antibody (dilution 1:100), nuclear staining was performed with ethidium bromide at 2.5 μg/ml and cell acquisition done with a flow cytometry (FACSCalibur, BD Bioscience), 5000 pRBC counted, the analysis was performed using FlowJo software. For analysis of surface cross-reactivity, pRBC incubated with the fluorophore-labelled secondary antibody only were used to define the non-reactive cell population; the percentage of positive cells was thereafter determined in all samples. Samples were considered positive for surface reactivity, when the percentage of positive cells was at least twice as high as the corresponding negative control; reactivity was scored as follows: low reactivity: 2-5× higher than control; medium reactivity: 5-105× higher than control; high reactivity ≥105× higher than control.

### Prediction of B cell epitopes

Prediction of B cell epitopes was carried out by submitting the protein sequences to Bepipred server [55]. Cut-off for posivity was set to 0.5.

### Peptide array

Peptide microarrays were manufactured by JPT (JPT Peptide Technologies, Berlin, Germany) with each slide contained three identical subarrays of a large set of overlapping amino acids of DBL1α-domains of five laboratory strains.

Slides were incubated for 16 h at 4°C in with 5 ug/ml of the antibody of interest in PBS buffer containing 3% of FCS and 0.5% of Tween (TPBS). After washing twice with TPBS and trice with distilled water, incubation with a Cy5-labelled secondary antibodies (Jackson ImmunoResearch) was carried out; followed by washing steps. Slides were scanned at 635nm using a GenePix 4000B microarray scanner (Axon Instruments, CA, USA) and images analyzed using GenePixPro 7.0 software in combination with the GAL file provided by JPT. The mean of fluorescence intensity obtained from the foreground and the local background were used to calculate the antibody responses; data presented here correspond to the average of the three subarrays.

## Results

### Expression of recombinant NTS-DBL1α in *Escherichia coli*

**Figure 1:**
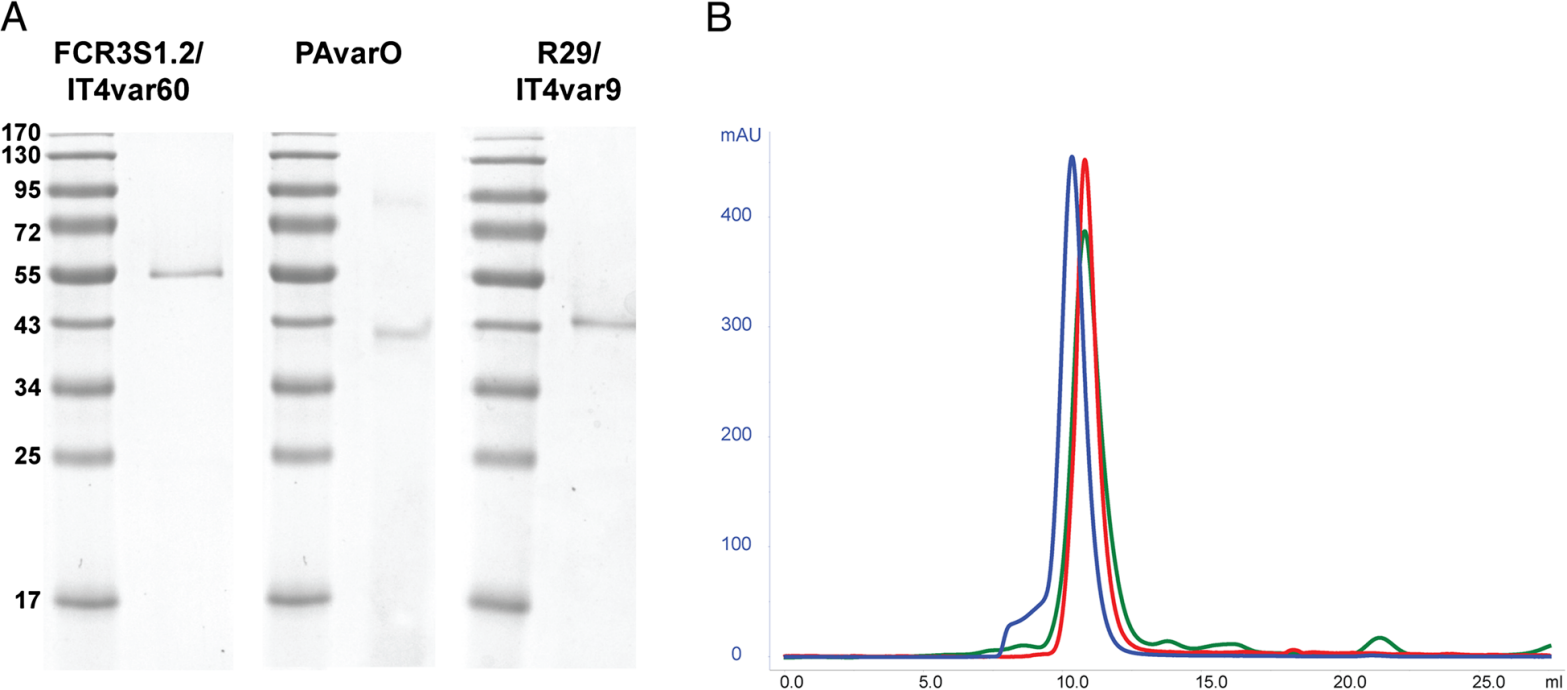
**Characterization of the recombinant NTS-DBL1α domains. A:** Recombinant NTS-DBL1α domains run on 12% SDS-PAGE gel and stained with Coomassie-Blue. Two μg of protein were run under non-reducing conditions. **B:** Size exclusion profiles of the recombinant NTS-DBL1α domains. The proteins were run on Superdex75 HR 10/30 to check homogeneity. IT4var60, PAvarO and IT4var9 are in blue, red and green respectively.

Recombinant NTS-DBL1α domains of group A rosette-mediating PfEMP1 IT4var60 [25], IT4var9 [19] and PAvarO [24] were expressed as hexa-His tagged recombinant proteins in *E. coli* (Figure 1A). Initial attempts resulted in insoluble proteins that aggregated in inclusion bodies. Proteins were subsequently refolded and purified, giving a final yield of 0.5 to 3 mg per litre. For NTS-DBL1α of IT4var60 further optimization trials were carried out by re-designing the domain boundaries and testing several expression strains. The construct spanning from amino-acids 1 to 481 expressed in *E. coli* Shuffle T7 express with overnight induction at 16°C gave the highest yield of soluble protein with about 10 mg per litre of bacterial culture. All protein ran at the expected size of monomer under non-reducing conditions and showed a single, gaussian shaped peak, when run on size exclusion column (Figure 1B).

### Antigenicity of distinct NTS-DBL1α domains in different animal species

**Figure 2:**
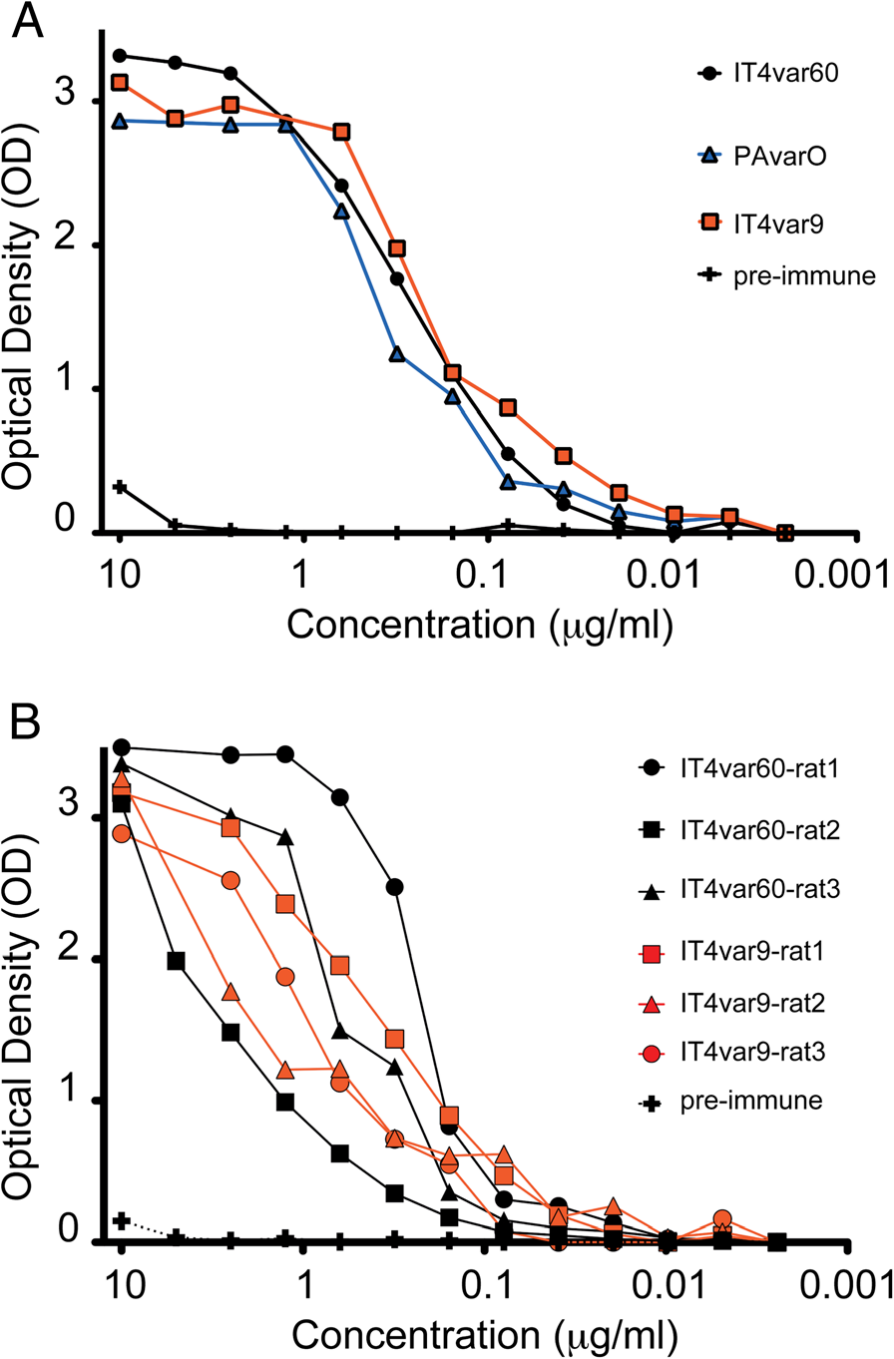
**ELISA titration of the generated antibodies.** Serial two-fold dilution of the generated antibodies in goat (**A**) or rat (**B**) towards the homologous NTS-DBL1α domains. Plates were coated with protein at 2 μg/ml and antibodies assayed at different concentration. For the rat sera the concentration was estimated assuming 10 mg of IgG in 1ml of serum.

To examine the levels of antigenicity of different recombinant NTS-DBL1α domains, associated with a rosetting phenotype, different animals were immunized and their IgG levels compared using ELISA. NTS-DBL1α_IT4var60_ and NTS-DBL1α_IT4var9_ were used to immunize one goat and three rats respectively, while NTS-DBL1α_PAvarO_ was used only for one goat immunization. All immunizations produced antibodies with a sigmoidal dose–response curve typically seen in antibody titration (Figure 2). Variation in antibody titres between animals of the same specie to distinct domains were minimal and probably not due to intrinsic differences in the protein immunogenicity by itself but rather to individual variation in the capacity of the immunized animal to generate immune response. In addition, no significant difference was detected in titres between animals immunized with shorter and refolded domains (PAvarO, ITvar9) *versus* the ones immunized with the longer construct, secreted as soluble protein in *E. coli* (ITvar60) (Figure 2).

Comparison of titres between different animal species revealed no difference in antibody titres towards the same protein. In order to compare purified IgG obtained from goats with rat sera a concentration of 10 mg of IgG per ml of serum in the immunized animal was assumed and the concentration calculated accordingly. It is likely that the individual levels are variable around this value, explaining the larger variation between different rats as compared to goats (Figure 2B).

In summary, all recombinant domains tested herein produced high titres of antibodies in immunized animals, suggesting that both rats and goats are good models to study immune responses to those domains, especially considering the low level of background seen with pre-immune sera/IgG.

### Antibody reactivity against native PfEMP1 displayed on the surface of parasitized erythrocytes

All antibodies were tested for their ability to recognize native full-length PfEMP1 expressed on the surface of pRBC. Parasites cultures were maintained as monovariant as previously described [24] and tested by flow cytometry for recognition by the antibodies generated.

**Figure 3:**
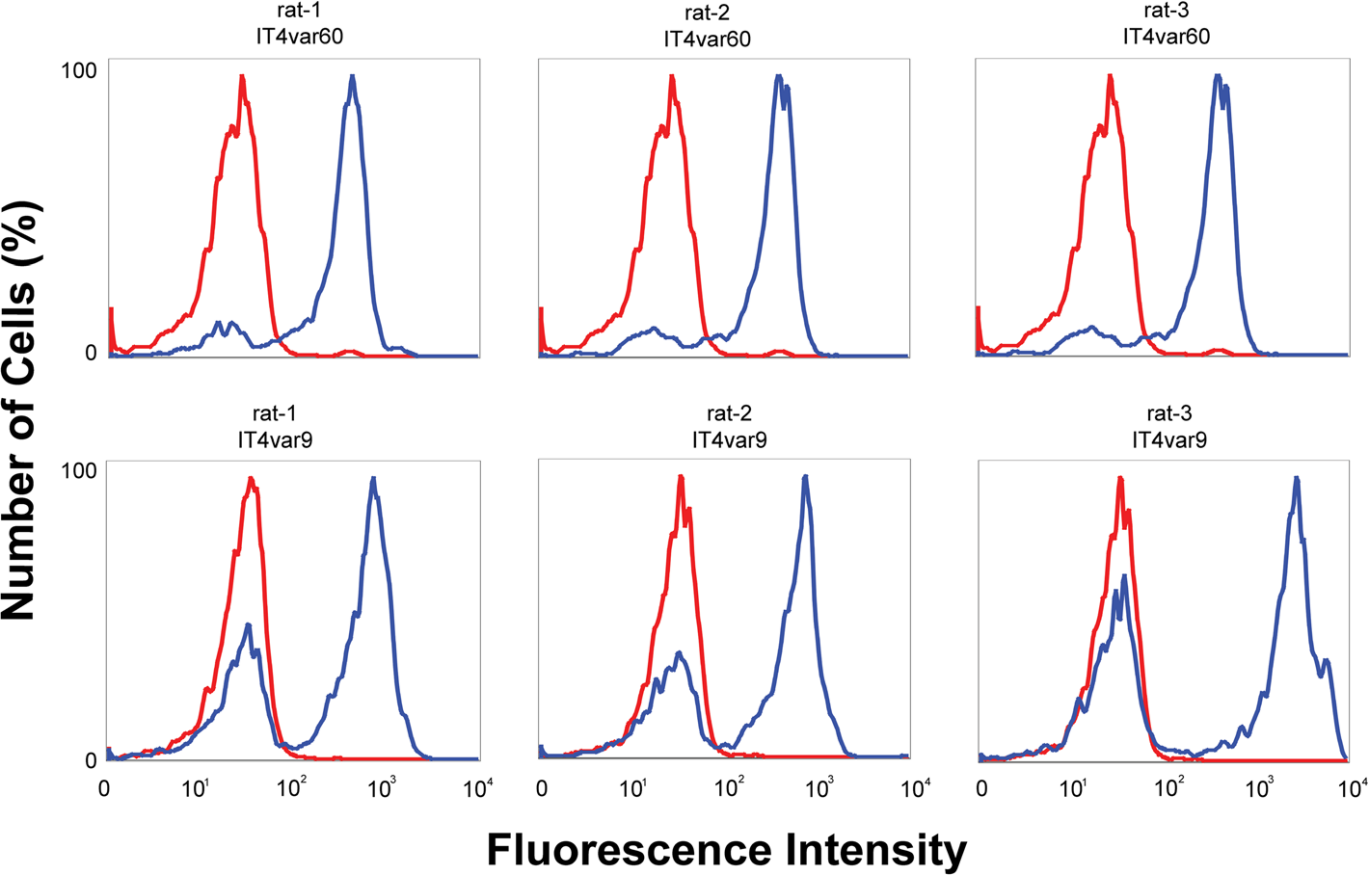
**Antibody reactivity against the native, surface expressed homologous PfEMP1.** Surface reactivity of rat sera with homologous pRBC as detected by Alexa488-conjugated secondary antibody and visualized by flow cytometry. Immune and non-immune controls are in blue and red respectively.

In line with the ELISA titres results, no difference was detected in the capacity to recognize the surface of pRBCs of homologous parasites between goat and rat antibodies (Figure 3 and [52]); all antibodies tested labelled the pRBC population expressing the homologous PfEMP1-variant. Further, there was no difference between Abs generated by immunization with shorter and refolded *versus* longer, soluble protein.

### NTS-DBL1α B cell epitope prediction

**Figure 4:**
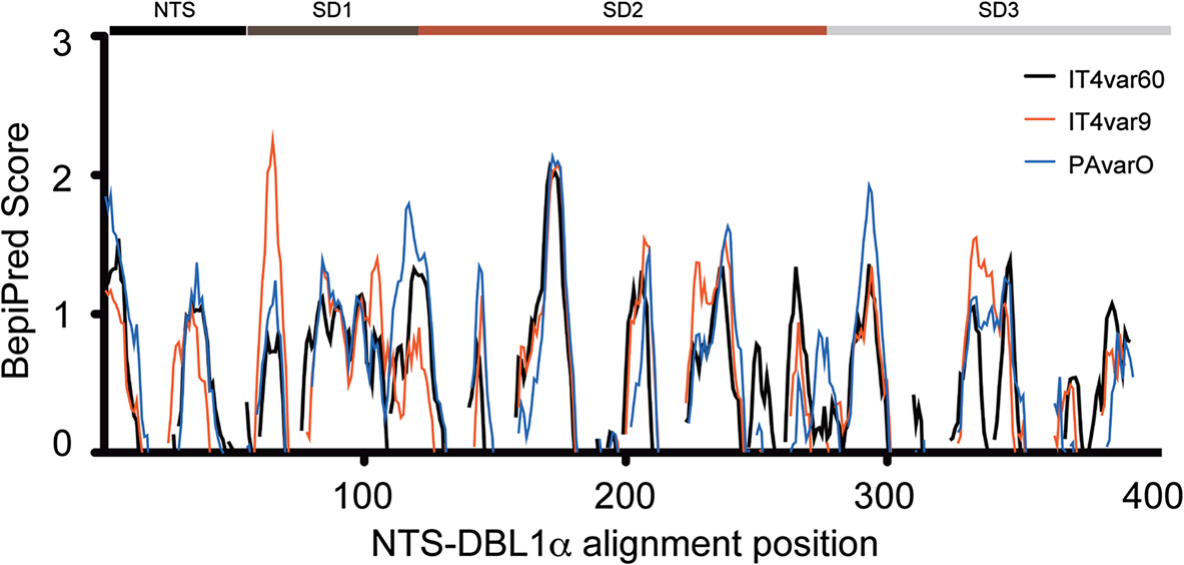
**B cell epitope prediction of the studied sequences.** B cell epitopes were predicted using the Bepipred server for the NTS-DBL1α sequences of IT4var60 (black), IT4var9 (red) and PAvarO (blue). Values above 0.5 are considered as predicted epitopes.

Epitopes exposed on the surface of the proteins and accessible to IgG were predicted using the BepiPred server [55]. For all three the proteins analysed 11 major antigenic areas were identified that were largely overlapping (Figure 4). Most of the predicted epitopes were spanning surface-exposed loop regions, according to the crystal structure of PAvarO and the molecular models of IT4var60 and IT4var9. The only epitope predicted to be targeting an α-helical structure is localized at the end of helix-7 of NTS-DBL1α, a region that has been previously shown not to be surface exposed on the full length PFEMP on the pRBC surface [52]. The loop of subdomain 3 (SD3), shown to be a target in anti-rosetting activity [52], is also consistently predicted as B-cell epitope.

### Mapping of epitopes by peptide array

Antibodies of all immunized goats and rats were tested on a peptide array holding five complete NTS-DBL1α sequences, in order to investigate homologous and cross-reactive responses in different animals. Each sequence was covered by approximately 100 15-mer peptides overlapping by four.

**Figure 5:**
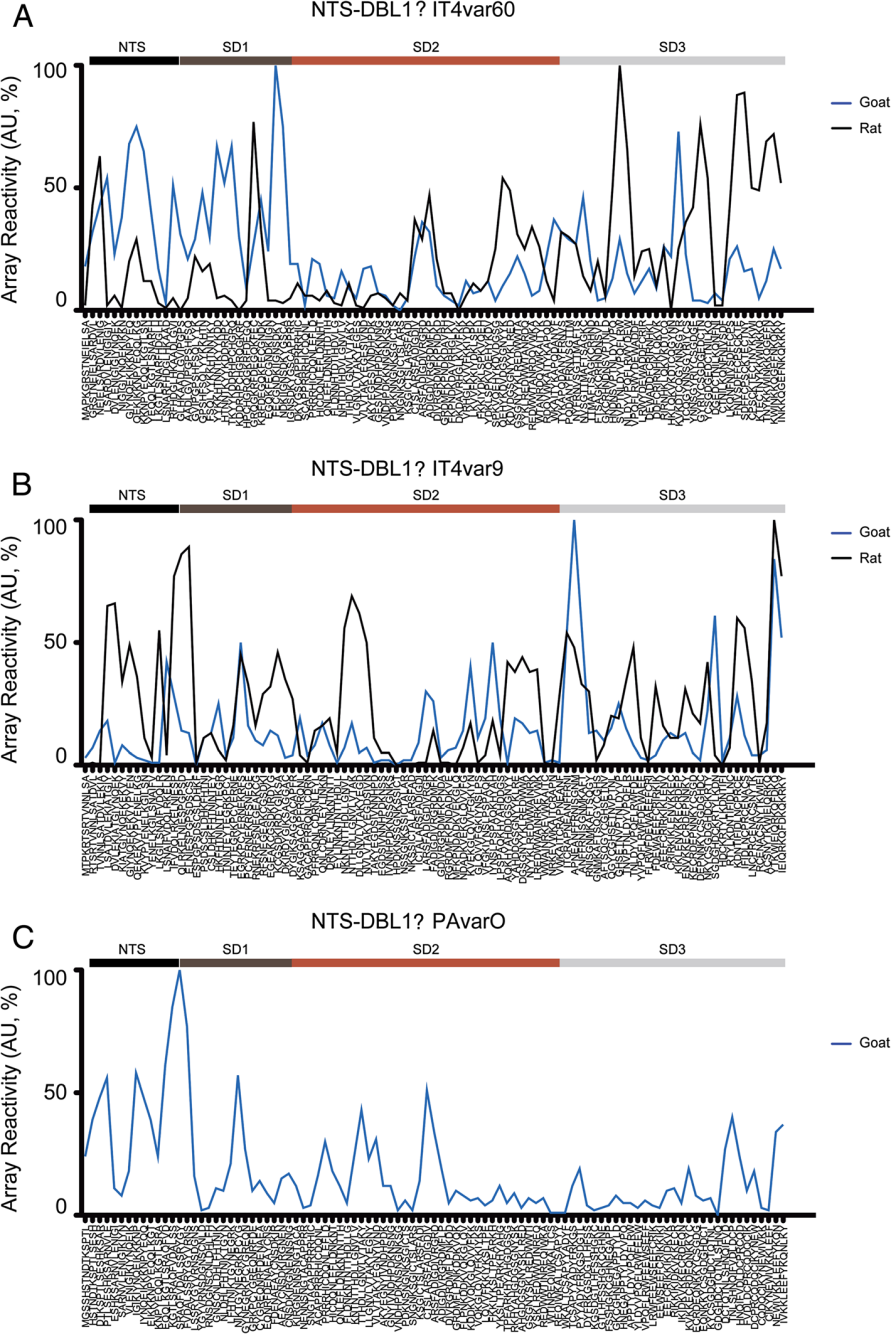
**Analysis of epitope recognition by peptide microarray.** Antibodies generated in goats (blue) and rats (black) were tested for peptide recognition against the homologous sequences on a 15-mers peptide array. For IT4var60 (**A**)*, IT4var9 (**B**) and PAvarO (**C**) goat IgG were tested at 1μg/ml while rat sera at 1:100 dilution. Shown is the average of the individual animals immunized with the same protein. Results are expressed as arbitrary absorbance units normalized to the highest value set as 100%. *Data for goat IgG are also presented in [52].

Firstly the responses towards the homologous sequences was analysed in order to identify converge and divergence in peptide recognition between different animal species. IgG reactivity of the goat was compared with average reactivity of the three immunized rats for IT4var60 and IT4var9 (Figure 5A-B).

For IT4var9, the reactivity of antibodies produced in rats was high for most parts of the protein; highest reactivity was found in the NTS and subdomain 1 (SD1) region of the molecule. The goat IgG showed a similar pattern of response with differences just in the amplitude of the response but not in the areas recognized (Figure 5B).

IT4var60 immunization showed a tendency of a higher response of goat IgG towards peptides localized in NTS and SD1 while rat antibodies responded better to the SD3 area (Figure 5A).

When comparing the reactivity of goat IgG obtained from immunization with distinct protein it was not possible to detect any consistent pattern in the epitope recognition (Additional file 1) with very different patterns between different animals. However, there was a general tendency that the N and C terminal parts of the molecules were more recognized by the antibodies.

Goat IgG was also analysed for the capacity of cross-recognizing peptides present on other proteins (Additional file 2). Very few peptides were cross-recognized, in line with the known difficulties of generating cross-reactive antibodies, all goats consistently recognized the conserved peptide LARSFADIG in all the NTS-DBL1α sequences tested. Immunizations with both IT4var9 and PAvarO generated antibodies that strongly recognized several sequences on the peptide array corresponding to the IT4var60 sequence: in particular, epitopes in SD1 and SD2 showed higher recognition by the IgG from PAvarO-goat as compared to the homologous IT4var60-goat (Additional file 2A).

### Cross-reactivity screening by ELISA and FACS

**Figure 6:**
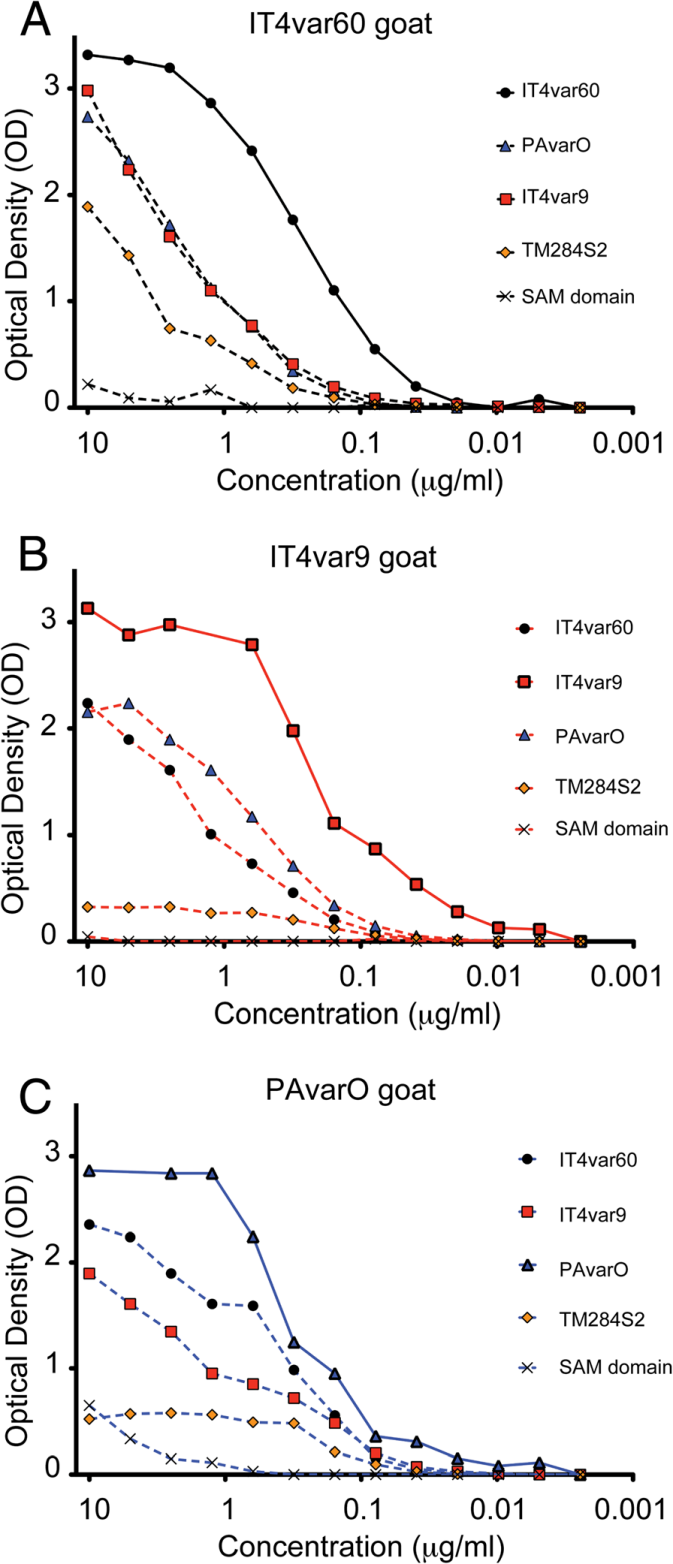
**Cross-reactivity of antibodies measured on the recombinant protein in ELISA.** IgG of goat immunized with IT4var60 (**A**), IT4var9 (**B**) and PAvarO (**C**) were tested for cross-recognition of heterologous recombinant NTS-DBL1α domains by ELISA. 2μg of recombinant IT4var60 (black dot), IT4var9 (red square), PAvarO (blue triangle), TM284S2 (orange diamond) or His-tagged negative control Sterile alpha motif (SAM) domain (black cross) were coated on the plate and antibodies were assayed at two-fold serial dilution.

**Figure 7:**
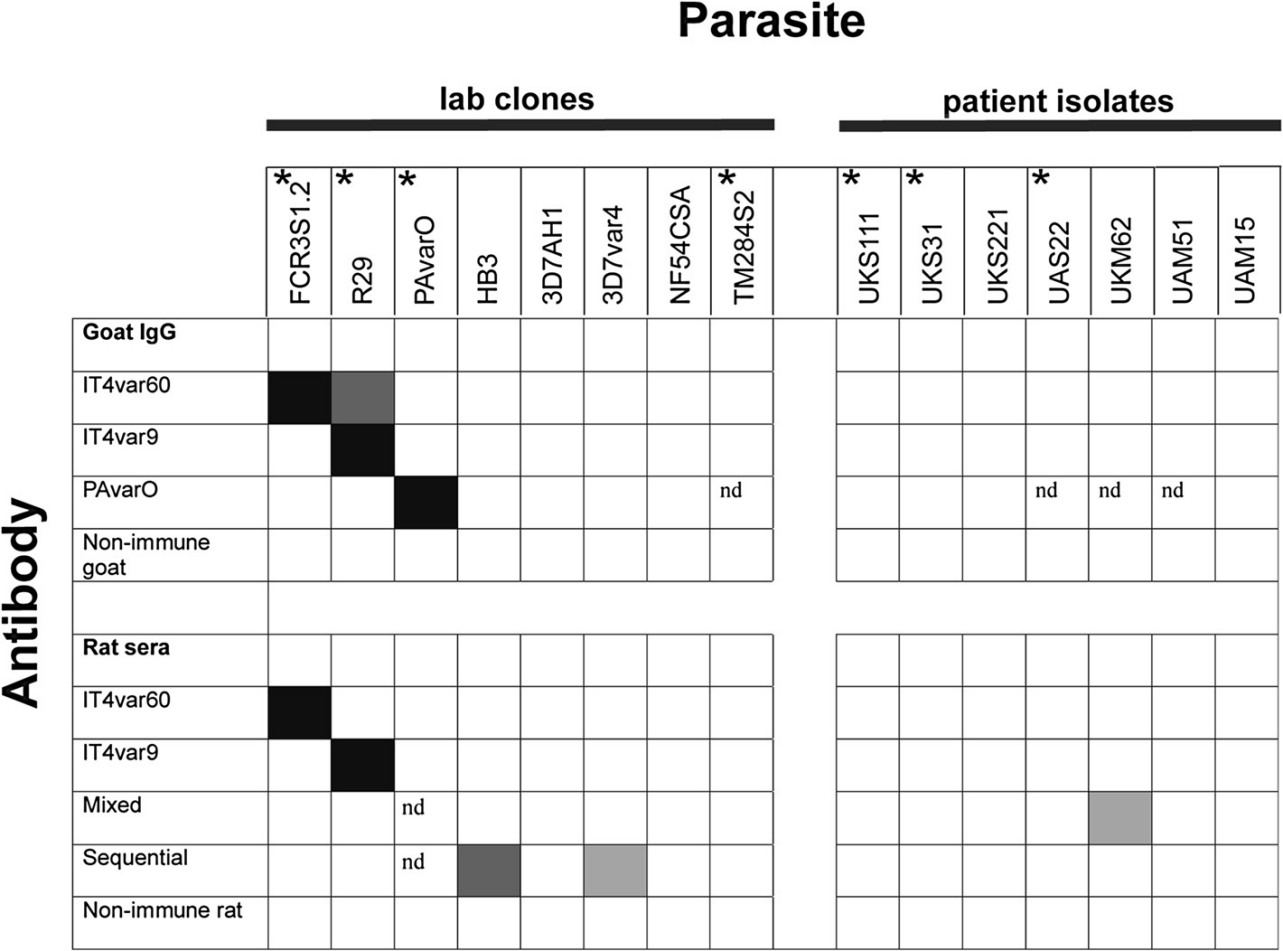
**Cross-reactivity screening of antibodies measured on native protein on the pRBC surface.** Analysis of surface reactivity of a panel of 15 parasite strains or isolates with goat IgG or rat sera generated against NTS-DBL1α-domains of IT4var9, PAvarO, DBL1α_S1.2var1, 3d7var5.2, PAvarO_ (mixed) and DBL1α_S1.2var1, 3d7var5.2, PAvarO_ (sequential). Empty squares: no reactivity; dark grey squares: reactivity of more than 10× above background; grey squares: reactivity of 5-10× above background; light grey squares: reactivity of 2–5× above background nd: not done. Star indicates parasites with a rosetting phenotype.

The generated antibodies were subsequently tested for their capacity to cross-react with heterologous NTS-DBL1α domains both by ELISA and by FACS (Figures 6 and 7). In ELISA cross-recognition of heterologous protein was observed in all immunized animals and titres of cross-reactive antibodies were high (Figure 6). Further cross-reactivity on linear peptides could be observed by analysis of the peptide array (Additional file 2). For example, goat anti-PAvarO IgG, which reacted with many peptides of IT4var60, had high recognition of the latter protein with a titre just slightly lower as compared to homologous protein (Figure 6C). Goat anti-IT4var60 IgG, on the other hand, showed some level of cross-recognition but with much lower titres as compared to homologous domain, as seen by the poor recognition of other peptides in the array (Figure 6A and Additional file 2).

Subsequently, the surface cross-reactivity of the antibodies was studied with pRBC of 15 different parasites including laboratory clones/strains and patient isolates. The antibodies were tested by flow-cytometry for their ability to recognize the surface of RBCs parasitized with heterologous parasite strains and correlated those data with ELISA and peptide-array data. Goat IgGs were used at 10μg/ml while rat sera was diluted 1:5 and 1:10. The homologous pRBC always displayed strong reactivity. However, binding to heterologous pRBC was observed only in a few combinations (Figure 7). Goat anti-IT4var60 displayed surface cross-reactivity with pRBCs of R29. Surprisingly the goat anti-PAvarO did not cross-react with FCR3S1.2, expressing IT4var60, despite the high number of peptides recognized in the array, suggesting that those specific area of the molecule might be hidden on full length PfEMP1. None of the rat sera generated against a single DBL1α-domain displayed surface cross-reactivity; however, low or moderate cross reactivity was observed against two laboratory and one patient isolate with rat sera against a mixture of three DBL1α-domains (Figure 7).

## Discussion

The NTS-DBL1α domain of the pRBC surface expressed PfEMP1 molecule has been shown to be of central importance for the virulence of the parasite. This domain is involved in rosetting [15,19,24,30] and has recently been shown to induce cross-reactive antibodies able to react with different rosetting-associated variants of the molecule [46], therefore being a promising candidate in the development of a vaccine against severe malaria. To efficiently design and test a vaccine it is important to understand if different NTS-DBL1α variants of the DBL1α1 subtype can induce consistent antibody responses in different animals. In this study, the response to immunization with distinct NTS-DBL1α domains in different animals was analysed employing ELISA assays, live cell surface reactivity, epitope prediction and peptide array.

PfEMP1-domains expressed by parasite strains with a rosetting phenotype [19,24,25] were chosen for expression of recombinant protein and animal immunizations. NTS-DBL1α constructs of PAvarO and IT4var9 covered aa 1–393 and refolded from inclusion bodies, for IT4var60 a longer construct was designed (aa 1–481), which was soluble in *E. coli.* All proteins were monomeric and folded as judged by mobility on non-reducing gel and size exclusion profile (Figure 1). There were no differences detected as both antibody titres and functionality was similar for the antibodies generated against the different constructs (Figures 2 and 3) suggesting that monomeric state and folding are sufficient requirements for potent induction of biologically active antibodies. Further, epitopes elicited by immunizations with different constructs were similar.

Previous studies showed induction of functional antibodies towards NTS-DBL1α domains in mice and rabbits [24,46,56] but no attempts has been made to compare induction of antibodies in different animals regarding the epitopes inducing or targeted by them.

All immunogens in the different animals, both species and individuals, elicited a similar response when analysing titres towards homologous proteins (Figure 2). This is in contrast to what was previously reported for DBL domains of the pregnancy malaria vaccine candidate VAR2CSA where substantial differences were detected when immunizing mice, rats and rabbits [57]. This could be possibly explained by similar induction of B-cell epitopes in different species, as well as conservation of immunogenic features in the proteins studied herein.

When comparing predicted and recognized epitopes substantial differences were found (Additional file 3). The prediction method used for this analysis identified mainly epitopes localized in the loop region of the molecule also predicted to be surface exposed. However, analysis of peptides that were in fact recognized revealed that also α-helical structures are predominant sites of immune responses. The conserved and non-exposed LARSFADIG sequence, present in SD2, was not predicted as highly immunogenic but antibody responses were frequent and high in all animals, suggesting also that conserved parts of the molecule are processed and presented on Major Histocompatibility complex (MHC) molecules. The observations presented in this study suggest that B-cell epitope prediction is informative; however, there might be a tendency for skewing towards surface epitopes that are present in unstructured loops. The analysis on epitopes recognized by immunized animals suggests that it is more common to have epitopes that span structured regions such as α-helices and that not necessarily are surface exposed. The latter could be a mechanism of immune evasion by which parasites direct the immune response towards epitopes that are not displayed on the cell surface and therefore impede the labelling of the pRBC with antibodies.

Analysis of the peptide array data for epitope recognition visualized substantial variation in between different animals, despite their isogenicity. No clear consensus was obtained when analysing different animal species immunized with the same protein (Figure 5). In addition, a limitation of this method is the fact that only linear epitopes can be detected; possibly, conformational epitopes are predominant and account for equal potency and efficiency of antibodies in different species. However, for both IT4var60 and IT4var9 there was a tendency in different species to recognize different epitopes in the N terminal part of the protein while more consensus was present concerning epitopes in SD3 of DBL1α.

In this study, surface labelling of heterologous pRBC with reagents against NTS-DBL1α-domains, did not reveal extensive cross-reactivity in heterologous parasite strains. Cross-reactivity in ELISA appears much more common for all three proteins analysed in the study. It has been suggested that coating of antigens to plastic surfaces as applied in ELISA-assays might unveil otherwise hidden epitopes [49,56]. When analysing epitopes cross-recognized by goat IgG the conserved motif LARSFADIG is consistently present. In addition, a peptide in the end of h7 is cross-recognized by some goat IgG. This part of the molecule has also previously been indicated as possible site for generation of ELISA cross-reactive antibodies [52]. Cross-reactivity in ELISA strongly correlates with the array cross-reactivity suggesting that most of it is due to those epitopes. These results should be taken into consideration when analysing cross-reactivity or sero-prevalence relying solely on ELISA data: despite the fact that the recombinant domain is correctly folded it might expose highly immunogenic epitopes that are not available in the full length PfEMP1 presented on the erythrocyte surface. Complementing ELISA with surface reactivity-data could minimize false positive results.

## Conclusions

This study compares different animal species for their response to immunization with distinct recombinant NTS-DBL1α domains of the DBLα1 subclass. All variants are able to elicit comparable titres of functional antibodies in all animal species here tested. Targeted epitopes of these antibodies are located in all subdomains of the NTS-DBL1α proteins and some of them map to the conserved, internal areas of the domain. Reactivity to epitopes on the homologous pRBC surface is strong; further, cross-reactivity between NTS-DBL1α-variants is common in ELISA and peptide array while weak and infrequent with the live pRBC surface of heterologous parasites.

The results show that NTS-DBL1α-domains display excellent antigenicity and are able to induce antibodies targeting adhesive events central to severe malaria in high titres. This suggests that a combination of distinct DBL1α-domains in a possible multimeric vaccine against severe malaria could overcome the problem of low cross-reactivity.

## Competing interests

The authors declare that they have no competing interests.

## Authors’ contributions

DA designed research, performed experiments, analysed data, and wrote the manuscript; LA performed experiments, analysed data; MW designed and supported the research, and wrote the manuscript; KM designed research, performed experiments, analysed data, and wrote the manuscript. All authors read and approved the final manuscript.

## Supplementary Material

Additional file 1 **Analysis of epitope recognition by peptide microarray.** Results as seen in Figure 5, but organized according to animal species, dividing goat (A) and rat (B) responses. IgG and sera were tested for peptide recognition against the homologous sequences, on a 15-mers peptide array, of IT4var60 (black), IT4var9 (red) and PAvarO (blue). Shown is the average of the individual animals immunized with the same protein. Results are expressed as arbitrary absorbance units normalized to the highest value set as 100%. Click here for file

Additional file 2 **Analysis of cross-recognition of epitopes on peptide arrays by goat IgG.** IgG of goat immunized with IT4var60 (black), IT4var9 (red) and PAvarO (blue) and non immune goat IgG (green) were tested for peptide recognition against the heterologous sequences on a 15-mers peptide array. Goat IgG were tested against NTS-DBL1α sequences of IT4var60 (A), IT4var9 (B), PAvarO (C), TM284S2 (D) and 3D7var4 (E). Results are expressed as arbitrary absorbance units normalized to the highest value set as 100%. Click here for file

Additional file 3 **Comparison of recognized *versus *predicted epitopes.** NTS-DBL1a sequences of IT4var60, IT4var9 and PAvarO with highlighted peptide recognized by immunized animals in peptide microarray (above a threshold of 30%) *versus* predicted epitopes (above a value of 0.4 from the Bepipred server). Peptides recognized by rat antibodies are coloured in orange, the ones recognized by goats in purple while predicted epitopes are in red. Red boxes indicate consensus recognition of the peptide by both rats and goats antibodies. Click here for file
