# Supplementary figures and images for: Analysis of antibody induction upon immunization with distinct NTS-DBL1α-domains of PfEMP1 from rosetting Plasmodium falciparum parasites
Source: Malar J. 2013 Jan 24;12:32. doi: 10.1186/1475-2875-12-32 (PMC3599323; doi:10.1186/1475-2875-12-32)

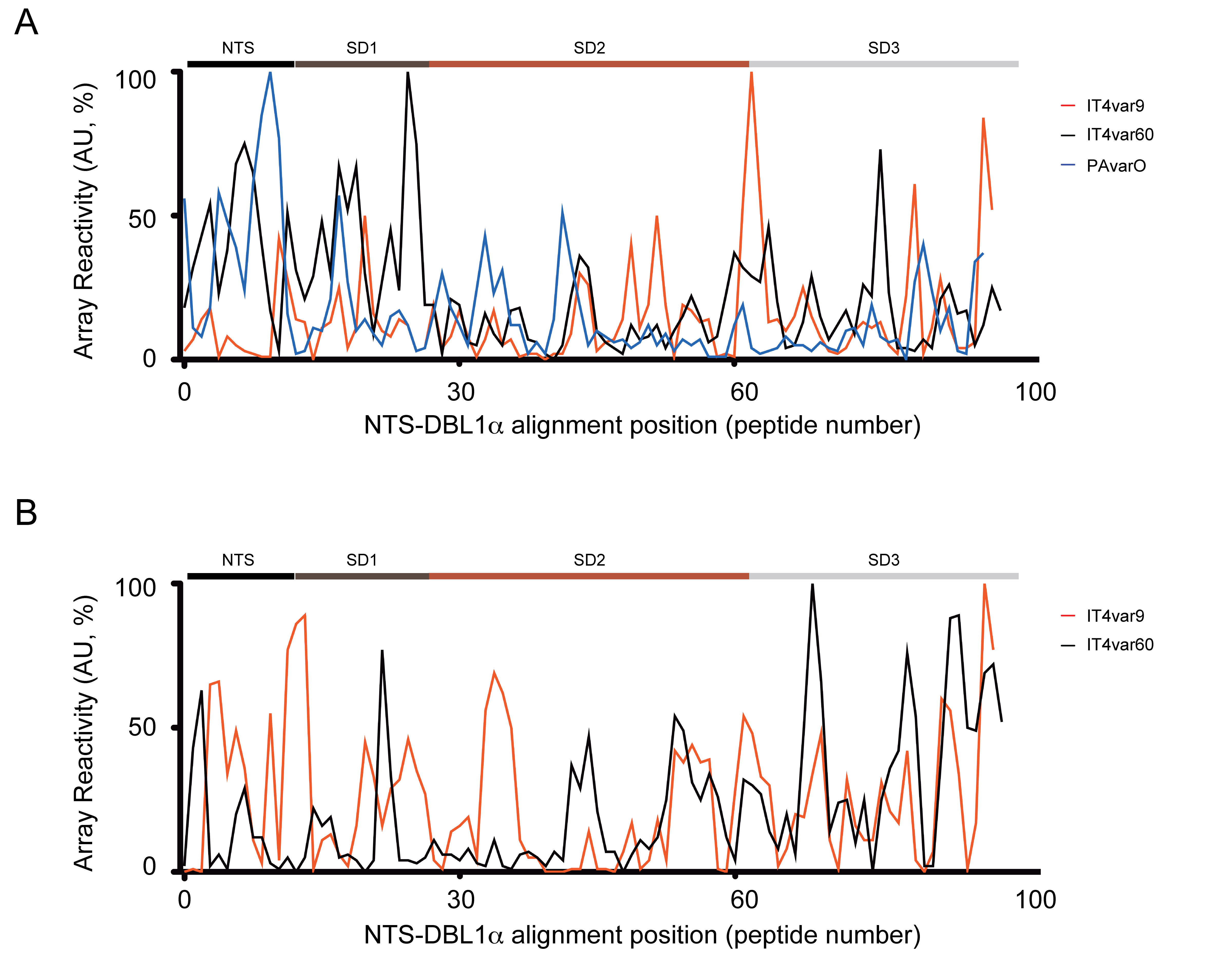

Supplement: Additional file 1 — Analysis of epitope recognition by peptide microarray. Results as seen in Figure 5, but organized according to animal species, dividing goat (A) and rat (B) responses. IgG and sera were tested for peptide recognition against the homologous sequences, on a 15-mers peptide array, of IT4var60 (black), IT4var9 (red) and PAvarO (blue). Shown is the average of the individual animals immunized with the same protein. Results are expressed as arbitrary absorbance units normalized to the highest value set as 100%. [file 1475-2875-12-32-S1.tiff]

A

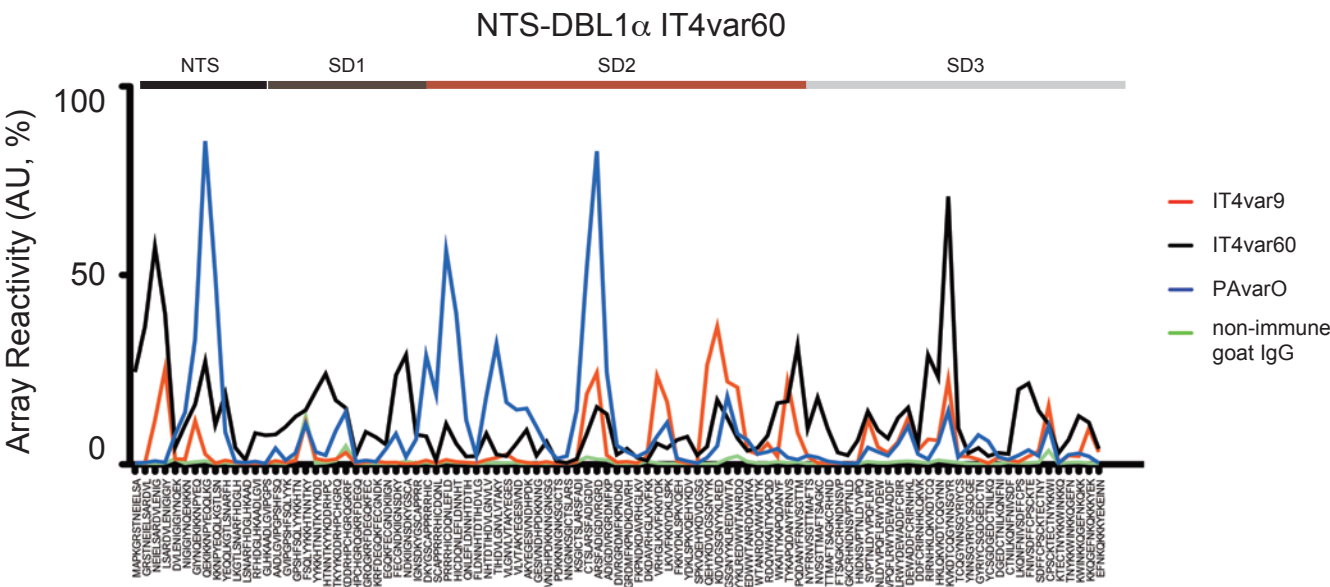

B

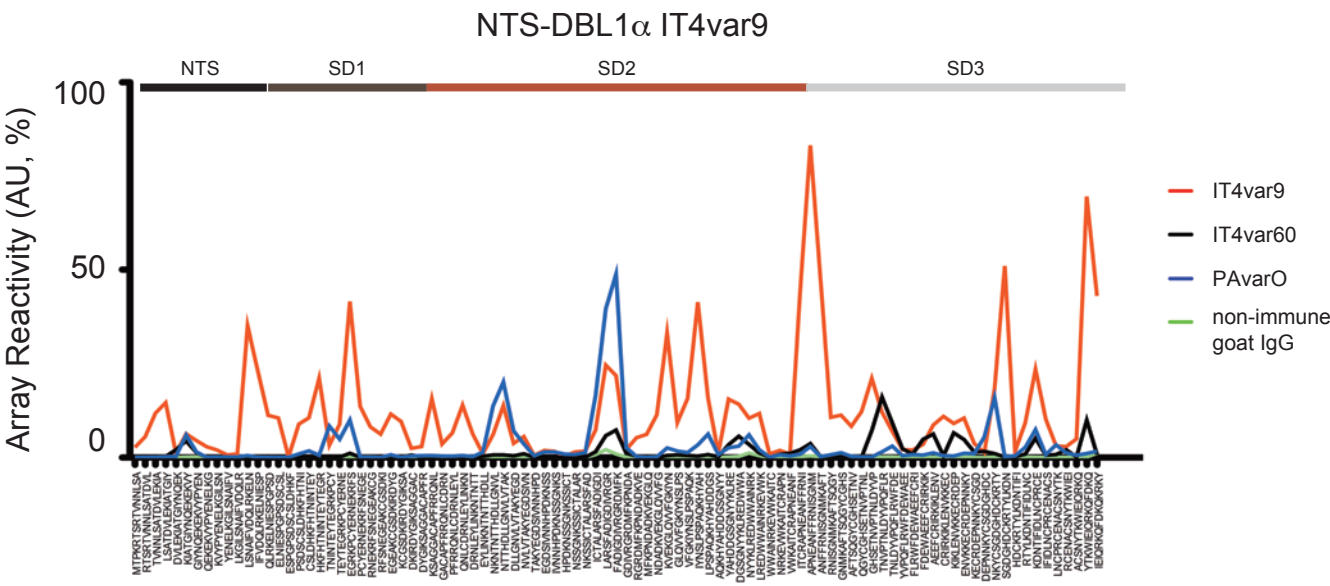

C

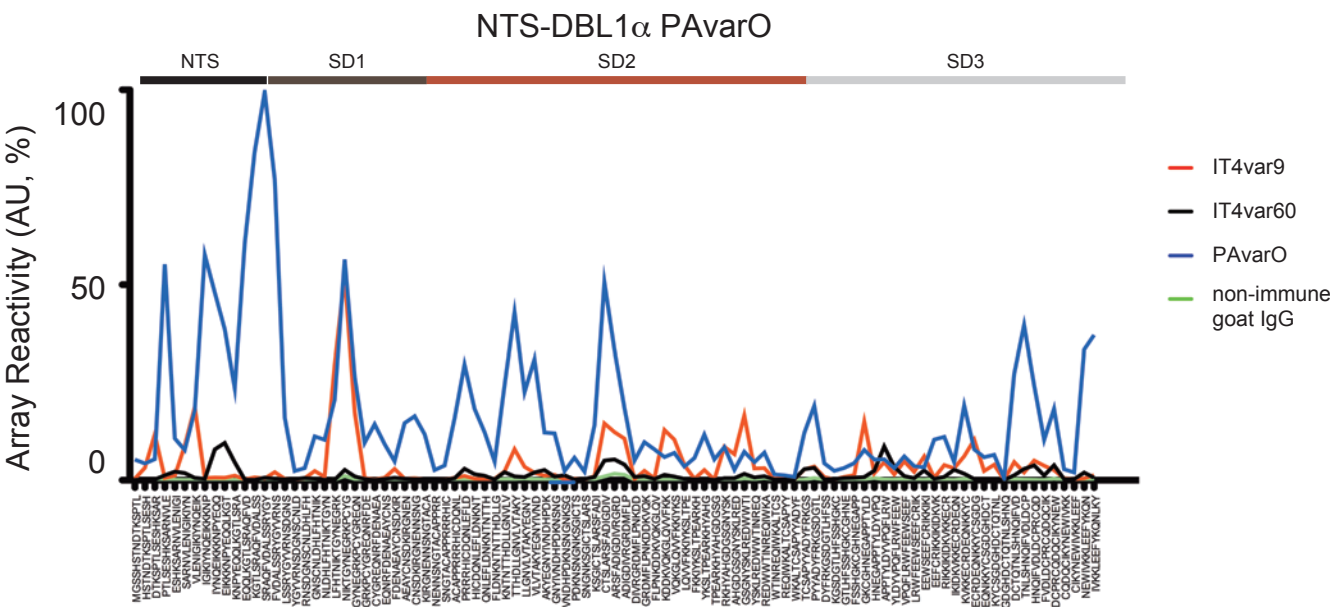

D

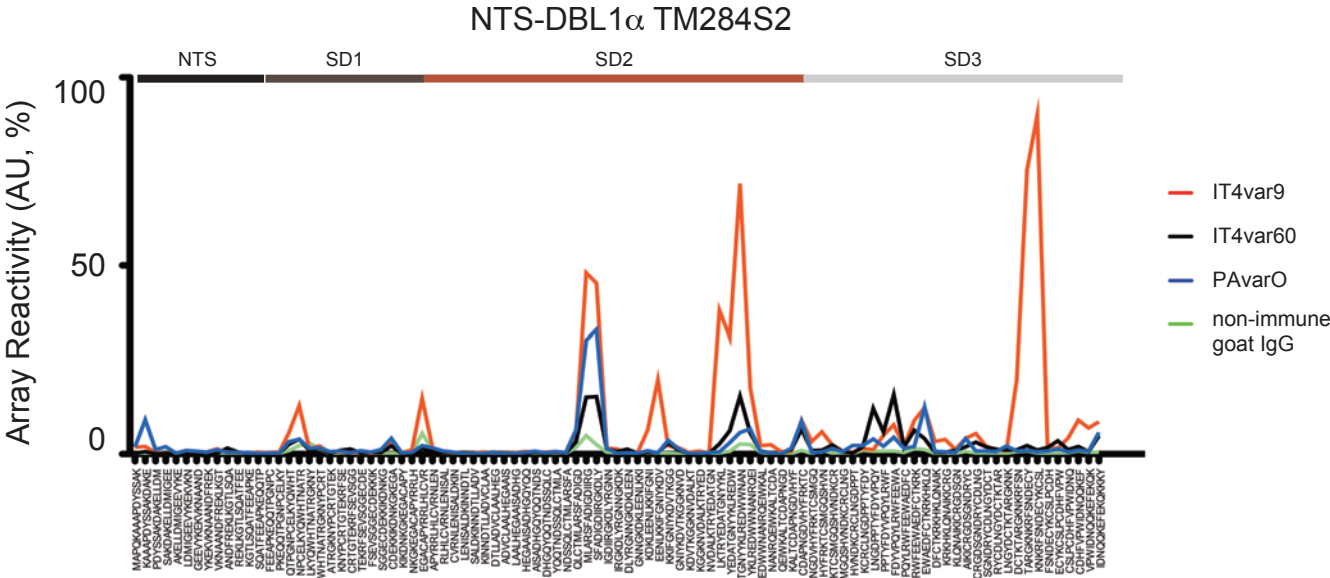

E

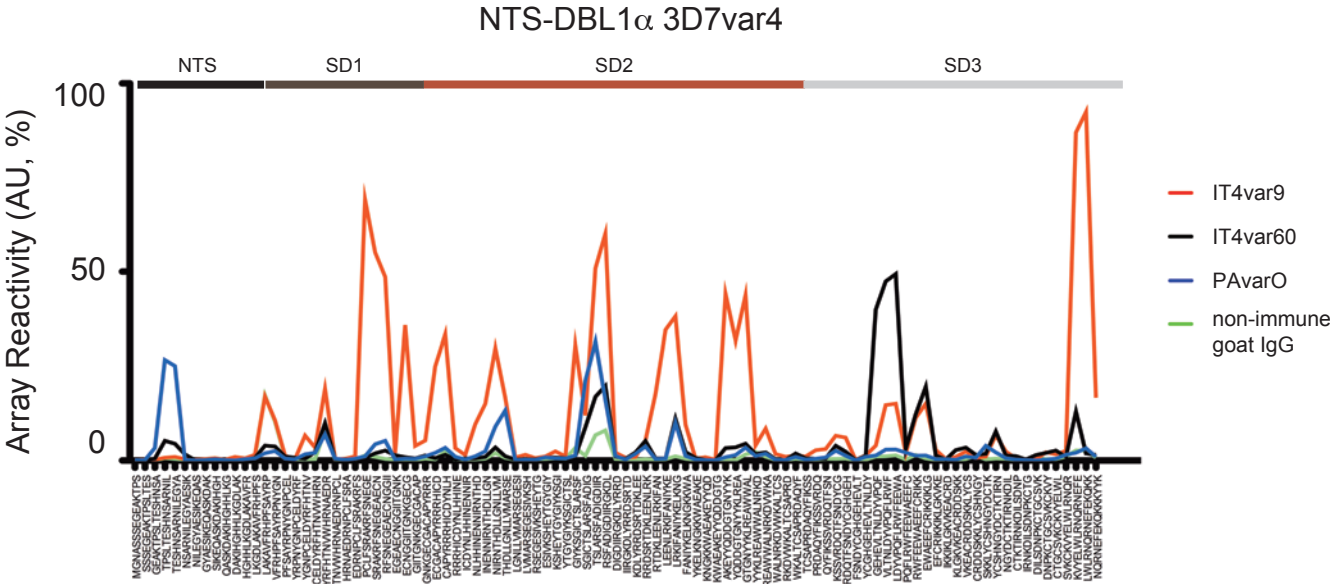

Supplement: Additional file 2 — Analysis of cross-recognition of epitopes on peptide arrays by goat IgG. IgG of goat immunized with IT4var60 (black), IT4var9 (red) and PAvarO (blue) and non immune goat IgG (green) were tested for peptide recognition against the heterologous sequences on a 15-mers peptide array. Goat IgG were tested against NTS-DBL1α sequences of IT4var60 (A), IT4var9 (B), PAvarO (C), TM284S2 (D) and 3D7var4 (E). Results are expressed as arbitrary absorbance units normalized to the highest value set as 100%. [file 1475-2875-12-32-S2.pdf]

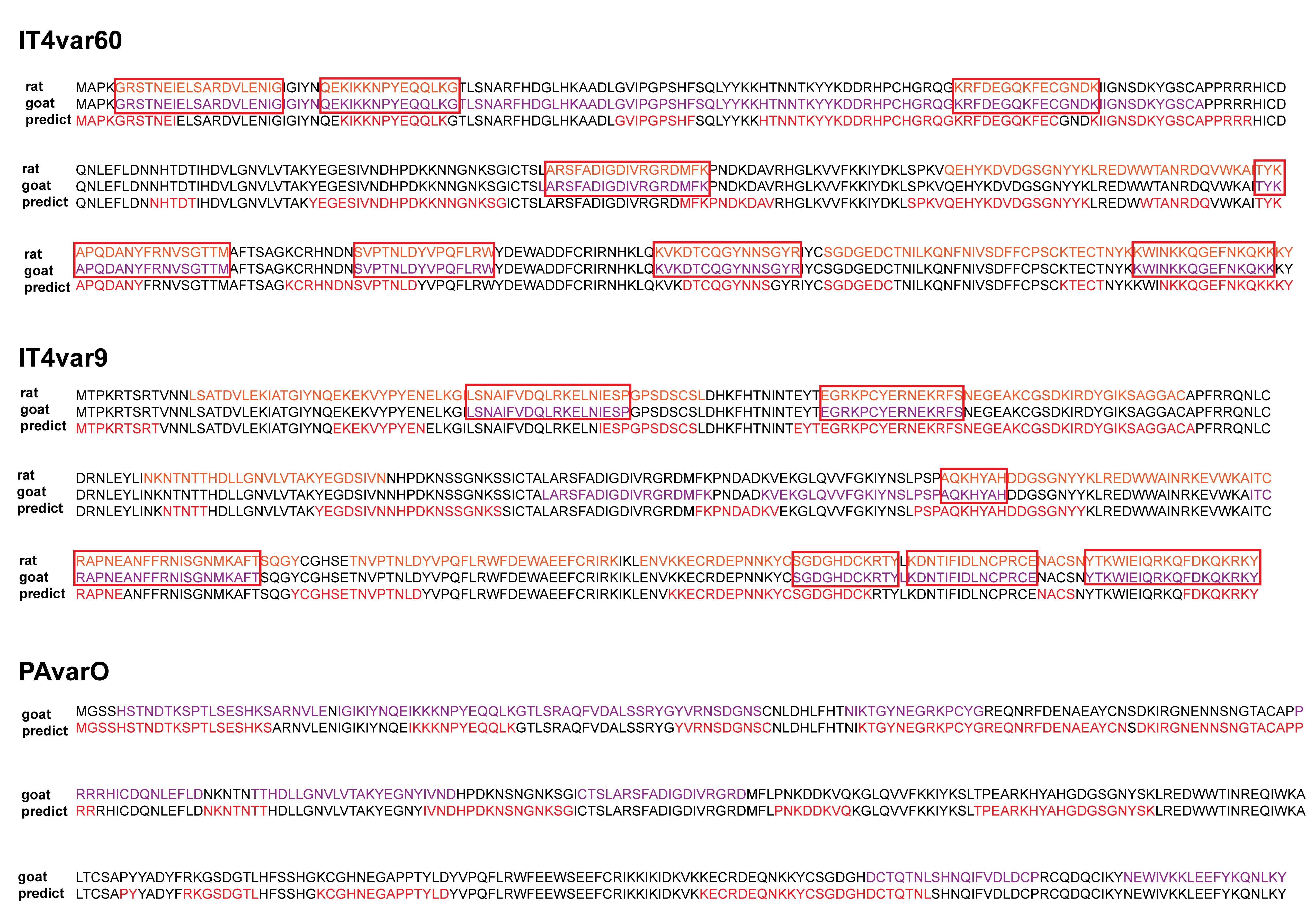

Supplement: Additional file 3 — Comparison of recognized versus predicted epitopes. NTS-DBL1a sequences of IT4var60, IT4var9 and PAvarO with highlighted peptide recognized by immunized animals in peptide microarray (above a threshold of 30%) versus predicted epitopes (above a value of 0.4 from the Bepipred server). Peptides recognized by rat antibodies are coloured in orange, the ones recognized by goats in purple while predicted epitopes are in red. Red boxes indicate consensus recognition of the peptide by both rats and goats antibodies. [file 1475-2875-12-32-S3.tiff]
